# Characterization of environmental and clinical surveillance inputs to support prospective integrated modeling of the polio endgame

**DOI:** 10.1371/journal.pgph.0004168

**Published:** 2025-02-07

**Authors:** Kamran Badizadegan, Kimberly M. Thompson

**Affiliations:** Kid Risk, Inc., Orlando, Florida, United States of America; Qatar University College of Medicine, QATAR

## Abstract

National, regional, and global poliovirus surveillance needs continue to expand and evolve. The 1988 global resolution to eradicate polio necessitated the creation and support for a global poliovirus surveillance system able to identify poliovirus transmission anywhere and everywhere. Clinical surveillance of patients that present with acute flaccid paralysis (AFP) became an essential tool, and the need for standardized laboratory methods to detect polioviruses isolated from stool samples of AFP patients led to the development of the Global Poliovirus Laboratory Network (GPLN) in 1990. Relatively recently, the GPLN expanded to include environmental surveillance to obtain additional information about poliovirus transmission in some geographies and to increase confidence about the absence of poliovirus transmission after successful eradication and/or the cessation of use of live-attenuated oral poliovirus vaccines (OPVs). Historical polio eradication strategic plans anticipated that successful global poliovirus eradication would lead to reduced requirements for financial investments for a poliovirus-specific surveillance system, and consequent transition of capacity and resources into integrated national disease surveillance systems. However, given the state of the polio endgame with ongoing transmission in several geographies, current global strategic plans include poliovirus-specific surveillance for the foreseeable future. In addition, the development and expansion of genetic testing technologies create new opportunities for poliovirus surveillance system designs. The expected growth (instead of decline) of poliovirus surveillance needs as of 2024, as well as innovations in laboratory technologies and expansion wastewater sampling, raise questions about the tradeoffs of different options and the future of poliovirus surveillance. This descriptive review of poliovirus surveillance evidence as of late 2024 aims to provide national, regional, and global decision makers with an understanding of prospective tradeoffs and uncertainties and to support prospective assumptions relevant for integrated policy, poliovirus transmission, and economic modeling for 2024–2035.

## Introduction

National, regional, and global poliovirus surveillance needs continue to expand and evolve with the development and evolution of national and regional polio elimination plans and the global polio eradication goals. Reliable surveillance data are an essential prerequisite for stopping poliovirus transmission, because success depends on knowing with high confidence where to intensify immunization efforts given that most polio cases occur in communities with low overall population immunity. In addition, formal certification of eradication of indigenous poliovirus transmission depends on achieving high confidence that no polioviruses are continuing to circulate while actively looking for them with high quality surveillance. Historically, poliovirus surveillance strategies evolved from national programs into regional and global plans.

### National

**Fig 1:**
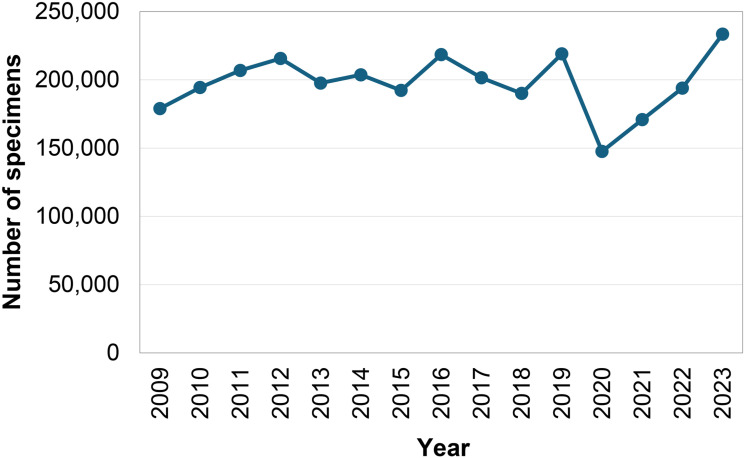
Annual number of AFP specimens reported by the GPEI.

National needs and objectives for poliovirus surveillance depend primarily on two factors: the extent of poliovirus transmission and the nature of different poliovirus vaccines in use. Prior to the introduction of poliovirus vaccines, countries mainly needed clinical surveillance to identify paralytic cases attributable to endemic transmission of wild polioviruses (WPVs) to support patient care and health resource planning. Following the introduction of poliovirus vaccines, and as WPV transmission and paralytic cases decreased, countries could begin to focus on using surveillance information to reactively respond to outbreaks with immunization campaigns and to preventively increase immunization coverage in under-vaccinated communities. In the US, the introduction of inactivated poliovirus vaccine (IPV) in the mid-1950s provided individual protection from paralysis for vaccine recipients, and polio incidence began to decline (see Fig 1 in reference [1]). With the introduction of oral poliovirus vaccine (OPV) in the 1960s, the use of live, attenuated vaccine strains not only conferred individual immunity, but also effectively competed with circulating WPVs in the population, resulting in population immunity to polio. As a result, the US and other countries that achieved and maintained high polio immunization coverage, locally eliminated WPV transmission. This demonstrated the potential for global poliovirus eradication, if and when all communities could contemporaneously achieve and maintain high coverage, and became the basis for the 1988 global commitment to eradicate polio by the year 2000 [2].

Unfortunately, along with its benefits, OPV use also came with risks of vaccine-associated paralytic polio (VAPP) and vaccine-derived polioviruses (VDPVs) [3,4]. In countries that stopped indigenous transmission of WPVs by achieving high coverage with OPV, rare cases of VAPP began to exceed the number of polio cases caused by WPVs and in some cases became the only reported cases. This motivated a shift in strategy to include IPV in the routine immunization schedules because IPV first (or exclusively) reduces (or can eliminate) VAPP. As countries with strong health systems achieved high routine immunization coverage and stopped poliovirus transmission and prevented VAPP cases, they began to rely on detecting and reporting rare cases of imported or locally transmitted polio through routine clinical care, such as the historical polio case workup [5] or the current acute flaccid myelitis workup in the US [6], which would detect polio as well as a variety of other paralytic illnesses.

Passive detection of rare polio cases, however, did not suffice to rapidly detect signals of poliovirus transmission in many countries, necessitating a program of active clinical surveillance. The process known as acute flaccid paralysis surveillance (AFP surveillance) since the 1980’s [7] continues to be a cornerstone of polio eradication activities and involves polio-specific clinical and laboratory workup of all children presenting with an acute paralytic illness. In addition, since diseases other than polio (e.g., Guillain-Barre Syndrome) also lead to clinical presentation of AFP, comparisons between AFP sample collection rates and expected AFP baseline rates provide a means to assess the quality of the AFP surveillance system for specific populations. AFP surveillance algorithms are designed to separate polio from non-polio AFP, without further delineation of the non-polio cases.

For OPV using countries that stopped WPV transmission but did not maintain high immunization coverage, the recognition of VDPVs led to expanded poliovirus surveillance through genetic analyses and other means to characterize VDPVs and to distinguish circulating VDPVs (cVDPVs, now sometimes called “variant polio”) and immunodeficiency-associated VDPVs (iVDPVs) from polio cases caused by WPVs and VAPP. Over time, some countries identified opportunities to perform environmental surveillance (ES) for polioviruses, which relies on collecting samples likely to contain human feces to test for evidence of poliovirus excretion within the population covered in the catchment area [8]. Countries develop(ed) these systems to benefit from early detection of importations from other countries and/or to monitor the die out of OPV-related polioviruses when making a national shift from using OPV to using IPV-only for routine immunization. Along with AFP surveillance, ES is now a cornerstone of polio eradication efforts.

### Regional and global

As polio eradication action and strategic plans evolved (reviewed in [9]), international coordination of poliovirus surveillance emerged as a pillar for regional efforts and the Global Polio Eradication Initiative (GPEI). Prior to 1988, the Americas demonstrated the use of AFP surveillance as essential in its regional polio elimination efforts [10]. In 1988, the World Health Assembly resolution to eradicate paralytic poliomyelitis (“polio”) created the need for global poliovirus surveillance able to identify poliovirus transmission anywhere and everywhere, for which it adopted the strategy of using AFP surveillance [11]. The GPEI included support for field and laboratory activities in its annual budgets for some geographies that lacked national capacity and required external financing [9]. The reliance on a standardized tissue culture-based method to detect polioviruses isolated from stool samples of AFP patients, as well as the philosophy that not every country needed the same polio testing capabilities, led to the development of a hierarchical network of predominantly small laboratories supported by a handful of specialized laboratories collectively known as the Global Poliovirus Laboratory Network (GPLN) [12]. The GPLN facilitates the development and deployment of field and laboratory methods, standardization and quality control, technical and other resource support, and information-sharing, as required for global eradication.

Following delays in achieving eradication and the global recommendation for all countries to include at least one dose of IPV in their national immunization schedules [13], the GPLN increasingly deployed and expanded ES systems to obtain additional information about poliovirus transmission in some geographies. ES relies on collecting samples likely to contain human feces to test for evidence of poliovirus excretion within the population covered in the catchment area [14,15]. The ES sample testing strategy essentially parallels AFP surveillance, and as of 2024 relies primarily on sample concentration followed by virus isolation using cell culture methods [14,15]. At the regional and global levels, ES provided an opportunity to observe and increase confidence about the absence of poliovirus transmission after successful eradication and/or the cessation of use of OPVs (e.g., [16]).

### Motivation for this descriptive research

Integrated poliovirus transmission, policy, and economic modeling (henceforth referred to as “integrated modeling”) of the polio eradication endgame includes prospective assumptions about the evolution of poliovirus surveillance. Although many studies use poliovirus surveillance data to perform statistical inference about past events or interventions (i.e., retrospective assessments) with or without short-term projections, only a relatively small number of prospective studies make assumptions about the future nature and performance of poliovirus surveillance systems (reviewed in [17,18]). Assumptions about the nature and quality of surveillance information play a critical role in these models because they simulate future signals that instigate the conduct of outbreak response supplemental immunization activities (oSIAs). In spite of significant changes in the scope and extent of poliovirus surveillance over the past few years, we did not identify any updated syntheses of prospective poliovirus surveillance-related inputs in our prior reviews [17,18] or subsequently or by other groups. Given the current status of polio eradication and endgame plans, including recent development of and updates to the global polio surveillance action plans [19,20], we aim to provide a descriptive review of the available evidence to learn from prior prospective assumptions for time periods that are now in the past, and to support prospective assumptions related to poliovirus surveillance in integrated modeling for 2025–2034 considering the most recent global polio eradication strategy [21].

## Methods

We use two sources of information to collect evidence about the quality and performance of poliovirus surveillance systems in recent years. These include (1) peer-reviewed literature related to the use of poliovirus surveillance information to control poliovirus transmission and certify its cessation, and (2) other literature produced by the GPEI and related entities (e.g., external advisory meeting reports, annual reports, surveillance reports and strategic plans). These collectively serve as the primary sources of observational data for this descriptive research as we seek to characterize the utilization (i.e., current and trends) of different types of poliovirus surveillance (i.e., AFP and ES) and performance of these systems.

Building on prior reviews of peer-reviewed poliovirus modeling studies by all GPEI-supported and independent modeling groups [17,18], we extracted and synthesized information to characterize the evolution of information about poliovirus surveillance in integrated modeling studies. We excluded retrospective statistical analyses of surveillance data (i.e., epidemiological studies), unless they played a critical role in informing prospective poliovirus transmission modeling studies. Although all modeling groups use poliovirus surveillance data, we acknowledge that the peer-reviewed literature relevant to this analysis primarily includes our studies due to our long-standing focus on prospective integrated modeling, which contrasts with the more diverse research emphasis of other modeling teams.

We extracted key poliovirus surveillance assumptions in prior prospective integrated modeling, including the time horizon, the poliovirus surveillance-related triggers used to initiate an oSIA, the time in days between the trigger and oSIA start, and the number of oSIA rounds. These surveillance-related characteristics determine programmatic performance with respect to stopping poliovirus transmission both in the field and in models. Notably, in contrast to statistical epidemiology which relies on retrospective data (and assumes that the past will reasonably represent the future for any prospective inferences), prospective integrated models use past performance as well as future plans to make prospective assumptions about performance for future time periods. Upon reaching the end of those periods, look back analyses can observe what actually occurred, learn, and motivate updates to prospective assumptions related to poliovirus surveillance for subsequent prospective model time horizons. Thus, review of successive prospective studies can document the evolution of poliovirus surveillance model inputs for integrated modeling made during a different prospective model time horizons. Importantly, we describe the inputs in prior integrated modeling that in anticipation of successful global poliovirus eradication assumed reduced requirements for financial investments for a poliovirus-specific surveillance system, transition of capacity and resources into integrated disease surveillance systems, and degradation of no longer needed surveillance quality over time. We also characterize the assumptions in peer-reviewed modeling studies that considered the anticipated quality of the information provided by poliovirus surveillance data in analyses of confidence of no circulation of poliovirus transmission to support certification of elimination and/or eradication.

Using available recent poliovirus surveillance data, we perform exploratory analyses to assess trends in poliovirus surveillance system utilization and quality. We retrieved the reported number of AFP specimens examined per year by the GPLN from published surveillance reports [22–33], and used information about reported cases released by GPEI each week [34,35] for WPV and cVDPV cases with onset during 2016–2023 (as reported through July 1, 2024) to characterize the distribution of the time in weeks of the official reporting of cases for each onset year. Since the results of this analysis reflect reporting only halfway through 2024, they include pending cases from earlier years (e.g., 2022 and 2023) that may still get officially reported in the future (i.e., some potential time-related censoring of the results for later years).

Since no comparable annual surveillance reports synthesize the details for ES, we extracted the annual number of ES specimens and the number of countries reporting ES from the GPEI Polio Information Systems (POLIS) as of July 1, 2024 [36]. (POLIS serves as a centralized database that includes polio case-based, environmental surveillance, and supplemental immunization activity data from all WHO regions, with limited access available under a Data Sharing Agreement.) Finally, similar to an analysis performed by Darwar et al. [37], we extracted data available from POLIS related to new emergences and the timing of associated oSIAs to analyze the time between onset of the first surveillance signal (i.e., paralysis of a case in a country without ongoing transmission of a new poliovirus strain or detection in ES) and the time in days to the implementation of the first oSIA round for emergences that occurred during each year for 2016–2023 as well as the total number of events.

## Results

We organize the results of this descriptive research into two sections. Section 1 divides our review of prior modeling publications related to poliovirus surveillance into three separate themes using subsections a through c. Section 2 presents the recent trends in AFP and ES activity, performance of reporting of surveillance detections, and characteristics of detections of new emergences and the time to the first associated oSIA based on data published by GPEI partners.

### Review of peer-reviewed studies

#### Prospective integrated modeling applications.

Detections of poliovirus transmission play(ed) a key role in triggering outbreak response in countries, and similarly in integrated modeling studies. As GPLN evolved over time, these triggers increased in complexity with respect to characterization of the nature (i.e., AFP, ES, iVDPV) and quality (i.e., ability to detect transmission) of surveillance information for modeling studies. Ideally, to support stratified global modeling that captures heterogeneity in immunization coverage and poliovirus transmission dynamics, model inputs should also reflect real variability in poliovirus surveillance information and its actual use for decision making. From a value of information perspective, poliovirus surveillance provides value if it improves overall outcomes (i.e., reduces expected cases by enabling earlier and/or more specific actionable insights that speed up outbreak response [38]).

Modeling studies generally use assumptions about surveillance information to characterize the detection threshold to signal the need for outbreak response (i.e., to initiate oSIAs). Studies published in 2008 modeled ongoing active clinical surveillance as “perfect AFP” assuming that AFP surveillance as implemented by the GPEI would detect the first case of any outbreak, compared to passive clinical surveillance that would detect an outbreak after some delay, modeled as detection of the fifth case [39,40]. Maintaining active clinical surveillance (i.e., high quality or near-perfect AFP surveillance) after polio eradication reduced the expected cases for the polio endgame substantially (i.e., by 75%) compared to passive surveillance, depending on the scenario [39]. In a comprehensive sensitivity analysis, the results showed that while active surveillance can increase the timeliness of outbreak response, it may come at substantial cost such that maintaining AFP surveillance long-term would not represent a cost-effective option [40]. The sensitivity analysis also included consideration of a hypothetical ES system that would detect the 5,000^th^ excretion of a given poliovirus, while noting the preliminary nature of the analysis given the absence of any ES system and lack of evidence on actual performance or costs [40]. The results of the ES analysis highlighted its expected increased value in settings that use IPV-only for immunization [40]. For example, in 2013, Israel detected local transmission of an imported WPV in its ES system, and it responded by conducting oSIAs [41].

We observed changes in the specific assumptions about the poliovirus surveillance-related oSIA triggers in successive integrated modeling studies [16,39,40,42–52], which we summarize in Table 1. The first column in Table 1 shows the earliest publication date of the study(ies) and associated references. The second column gives the associated model time horizon. The third and fourth columns provide the AFP and ES triggers used, while the fifth column gives the time since the detected trigger and the start of oSIAs, and the sixth column gives the number of oSIA rounds applied in the model, all of which occur 30 days apart.

**Table 1:** Prospective model assumptions about the time between case detection and start of oSIAs.

Date [ref]	Time horizon	AFP case detection trigger base case (alternatives)	ES detection trigger base case (alternatives)	Time in days between oSIA trigger and start base case (alternatives)	Number of oSIA rounds
2008 [39,40]	2010–2029	1 (5)	NA	70 (45)	3
2015 [42], 2016 [43]	2013–2052	1	NA	45 (30 or 50)	4 (or 6 in high-transmission subpopulations)
2020–2021 [44,45,48,49]	2019–2023	Depends on time period and income level 1970–2018: 1, 2, or 3 2019–2024: 3, 4, or 5 2025–2027: 5–13 2028–2058: 5–30	Approach described based on [16], but not used until 2021 when ES signals began to trigger oSIAs	Until 2016: 60 2017–2058: 45	Until 2016: 3 2017–2058: 2
2022–2023 [46,47,50,51]	2022–2026 or 2035	Depends on time period and income level 1970–2018: 1, 2, or 3 2019–2024: 3, 4, or 5 2025–2027: 5–13 2028–2058: 5–30	Depends on time period and income level, if ES exists in the modeled subpopulation, methods based on [16]	45	2
2024 [52]	2024–2035	2024–2035: 3, 4, or 5	Depends on time period and income level, if ES exists in the modeled subpopulation, methods based on [16]	45	2

Studies published in 2015 revisited polio eradication endgame strategies following the release of an updated polio eradication strategic plan [53]. These studies explicitly stratified the global population into heterogenous blocks and subpopulations to simulate variability in immunization policies, income levels, poliovirus transmission dynamics, risks, and surveillance assumptions [42,54]. For the run-up period (i.e., the time that precedes the prospective modeling time horizon), implementation of oSIAs occurs in the model subpopulations with approximately the same timing, number of rounds, and delay as occurred, which can occur due to the known timing of actual outbreaks and oSIA characteristics. The prospective modeling studies published in 2015 [42,54] focused on identifying strategies that would increase the probability of a successful polio endgame, including risk management of reintroduction risks from iVDPV excreters, containment breaches, and unexpected use of OPV after OPV cessation. With respect to surveillance, the modeling assumed subpopulation-specific detection thresholds of either 1, 2, or 3 polio cases (i.e., confirmed by AFP surveillance) as the trigger for outbreak response, and it did not include any degradation of AFP surveillance quality over time or consider any information from ES [42]. Additional modeling of outbreak response characteristics [43] reinforced earlier modeling results that identified the importance of rapid, high coverage, and sufficiently large and numerous oSIAs and the necessity of rapid detection [38].

As the GPEI invested substantially in expanding ES into selected low-resource geographies [19,20], modeling studies began to characterize the potential for ES detections to trigger outbreak response (as well as its contribution to confidence of no circulation, as described below). A review of poliovirus ES studies published prior to 2017 identified considerable variability in system designs and uncertainties about costs and performance [55]. As a result, with respect to characterizing the potential performance of ES as supported by GPEI expansion efforts, modeling studies considered the experience with ES in Pakistan and Afghanistan to explore different detection threshold options for both AFP and ES [16].

Following the observation of the actual implementation of risk management strategies after 2016, updated integrated modeling began to transition from assuming the prospective implementation of model-recommended and ideal strategies [56], to prospectively modeling outcomes based on retrospective actual performance and experience [44]. The surveillance assumptions included values for ES quality coefficients that correlated AFP detection threshold assumptions for 2018 for modeled blocks and subpopulations with ES quality (for countries with both AFP and ES) [44]. Modeling updates in 2019 [44] found that during 2016–2018, delays in oSIA responses implied the need to shift the triggers for oSIAs from occurrence of the first case (in the model) to the second or third modeled case (as indicated in Table 1). In addition, with oSIAs not occurring as aggressively as originally envisioned (i.e., not as fast, not as large in scope, or not with relatively high coverage) as required for success in polio eradication in modeling published before March 2016 [42,43]. The 2019 model update [44] also made adjustments to assume prospectively worse oSIA performance, with the retrospective implications of this shift explored in a look back analysis [57]. Based on published polio endgame plans that included transitioning away from reliance on polio-specific resources [58], the model also assumed that external support for AFP surveillance would end after OPV cessation, and that AFP surveillance quality would decline over time [44]. This modeling included updated analyses for iVDPV risks [59] and other risks (i.e., unexpected OPV use after homotypic cessation [60]), and it particularly focused on the inadequate performance of oSIAs, which failed to stop outbreaks [45].

The global experience with the COVID-19 pandemic and GPEI ramp downs in financial support that occurred in some geographies between 2020–2023 also contributed to the degradation of both surveillance quality and oSIA performance. In 2020, disruptions caused by the COVID-19 pandemic led to some delay of oSIAs, although reduced population mixing and less international travel helped to control the spread of outbreaks [48]. For 2021–2023, novel OPV2 (nOPV2) strains became available in limited quantities for oSIAs, and modeling studies identified national and regional decisions to delay oSIAs to wait for nOPV2 as a key source of delay that contributed to the longer times shown in Table 2 for these years [47,49]. Modeling studies continued to highlight the poor quality of SIAs (i.e., late, low-coverage, insufficient in scope) as a key failure mode for the polio endgame [17]. Several studies highlighted a variety of additional factors that likely contributed to these delays, including delays in shipping specimens to laboratories and in laboratory confirmation, particularly for cVDPV cases, and insufficient supply and delayed delivery of vaccines, particularly nOPV2 [37,61–66]. Reviews of the experience with the timing of oSIAs during periodic integrated model updates suggested that the prospective assumptions for 2019–2024 [44–51] led to modeling inputs consistent with observed experience. Thus, integrated modeling studies published during 2020–2023 (e.g., [46–51]) that included the 2018 assumptions about the degradation of poliovirus surveillance quality over time [44], performed reasonably well, albeit for different reasons than initially assumed (e.g., COVID-19 and programmatic defunding instead of successful polio eradication).

**Table 2:** Number of days until the first outbreak response supplemental immunization activity (oSIA) after the onset of paralysis for an AFP case caused by any wild poliovirus type 1 (WPV1) and/or circulating vaccine-derived poliovirus (cVDPV) of any type. After 2020, positive detection of a cVDPV by environmental surveillance (ES) used as the starting date, if ES detection occurred earlier than the first AFP case or if oSIAs began based on ES detection alone (without detection of an AFP case).

Signal year*	Number of new outbreaks with oSIAs	Days from signal to first oSIA
2016	3	33
2017	3	120
2018	9	71
2019	53	93
2020	43	201
2021	32	268
2022	28	188
2023	14	192

*Transmission signal refers to AFP onset before 2021 or to the earlier of AFP onset or a positive ES detection for 2021 on).

Recently published integrated modeling studies that explored globally coordinated cessation of bivalent OPV (bOPV) in 2027 suggested substantial increases in expected annual polio cases for 2027–2035 [46,51]. A 2024 study that revisited this analysis recognized the contribution of the surveillance assumptions in the integrated model, particularly the assumed degradation in surveillance quality after 2025, which limited the ability to control outbreaks [52]. To remove the effects of surveillance quality degradation, this study assumed any further surveillance quality degradation (relative to current surveillance quality) would only begin after the model time horizon (i.e., after 2035) [52].

Actual experience with type 2 cVDPV importations demonstrated the key role that wastewater surveillance played in the detection and monitoring of transmission in 2022–2023 in the US [67,68], the United Kingdom [69], Israel [70], and Canada [71], in some cases building on systems developed for SARS-CoV-2 surveillance. The detections of sustained poliovirus transmission of cVDPVs of different types in countries that use IPV-only shows that immunization responses to these events occurred even in the absence of cases (e.g., the United Kingdom, Israel) or with detection of a single polio case (e.g., the US, Israel) [41,68,72,73].

As of the end of 2024, integrated modeling assumptions reflected numerous major shifts in the polio eradication endgame relevant to the nature and quality of prospective poliovirus surveillance for 2024–2035 [52]. First, with the introduction and widespread adoption of IPV in routine immunization, the time between cases increases due to the protection of some individuals from paralysis offered by IPV, which may increase the time for starting oSIAs in subpopulations for which detection and implementation of an oSIA depends on the observation of one or more AFP cases. Second, recent experience of immunization responses to poliovirus detections in IPV-only countries shows that the assumptions related to the decline of surveillance made in prior prospective integrated modeling did not: (1) anticipate the detection of paralytic cases from poliovirus transmission in IPV-only countries and (2) unrealistically delayed response to the time of the detection any such event to the fifth case, if it ever occurred. We emphasize that relatively higher-income countries using IPV-only with high immunization coverage do not and will not likely contribute substantially to global transmission, and the earlier assumptions did not matter in the past and only matter prospectively due to unsuccessful eradication, failed OPV cessation, and increased concentration and movement of un(der)-vaccinated individuals. Third, delays in achieving the goal of polio eradication imply the need for continued poliovirus surveillance and a prolonged polio eradication endgame, with the GPEI partners continuing to discuss plans and budgeting through 2029.

#### Economic analyses of poliovirus surveillance.

Over the past two decades, several economic analyses explicitly provided insights about the value of poliovirus surveillance in supporting polio eradication and the endgame. A 2006 study of the GPLN estimated global costs of $21 million for the laboratory component of poliovirus surveillance, which represented 17% of an estimated approximately $125 million for poliovirus surveillance overall (i.e., including sample collection, management, and all non-laboratory surveillance activities) [74]. With overall expectations around that time of global incremental net benefits (INBs) of $40–50 billion (US$2002) for 2010–2029 if the world achieved polio eradication and successfully ended poliovirus vaccine use [39], surveillance represented a minor cost.

More recently, a 2015 economic analysis of the best modeled potential polio eradication endgame estimated approximately 16 billion (US$2013) for INBs during 2013–2052, while highlighting uncertainty about prospective risks and their management [42]. Specific to poliovirus surveillance, a 2017 study estimated that in a world with successful risk management of cVDPVs [42], if iVDPV risks emerged as substantial and researchers succeeded in developing a highly-effective poliovirus antiviral drug, then proactive screening to identify and treat iVDPVs could provide INBs of approximately 0.7–1.5 billion (US$2013) [75]. A 2019 study surveyed the GPLN and estimated $43 million (US$2016) in laboratory costs for poliovirus surveillance [76]. In 2021, a study of poliovirus surveillance in Pakistan and Afghanistan anticipated annual resource requirements of tens of millions of dollars (US$2019) until eradication and depending on the types of surveillance includes (i.e., AFP, ES, lot quality assurance sampling (LQAS)) [77].

The yet unfinished and uncertain polio eradication endgame as of mid-2024 [9], and availability of innovations in laboratory methods as potential alternative approaches to process AFP and ES samples [78–84], lead to an increasing focus on the effectiveness, health economics, and trade-offs of polio surveillance options over the next decade and beyond. Continued and expanding investments in poliovirus surveillance provide an indication of expected growth (instead of decline), which raises questions about the economic and other tradeoffs of different options and the future of poliovirus surveillance overall [20,58].

#### Certification of eradication of indigenous poliovirus transmission applications.

Poliovirus surveillance information also plays a key role in modeling the confidence that successful eradication has actually occurred. The Americas relied on a retrospective statistical analysis of surveillance data to support certification of the eradication of indigenous WPV transmission for the region in 1994 after 3 years with no detected WPV cases by active AFP surveillance [85]. However, theoretical stochastic modeling performed to support the certification deliberations also characterized the probability of silent or undetected WPV transmission as a function of time since the last case [86]. This modeling provided additional support for the 3-year period by suggesting high (i.e., 95%) confidence of no circulation given the observation of no detected cases 3 years after the last case assuming perfect AFP surveillance [86].

Since 2012, multiple studies performed similar stochastic modeling to characterize the confidence of no circulation for hypothetical populations with different characteristics [87], including those with an under-vaccinated subpopulation [88], and for specific populations (i.e., Israel [41,89], Tajikistan [89], northern India [89], northern Nigeria [89,90], Pakistan and Afghanistan [16,91–93], and New York (for an outbreak caused by an imported type 2 cVDPV) [68]. Notably, the studies of specific populations explicitly considered the imperfect information provided by poliovirus surveillance and immunization activities for those countries. An August 2015 study that applied a different approach suggested very high confidence of no WPV transmission in Nigeria with perfect surveillance and slightly reduced confidence (i.e., 93% expected by the end of 2015) with 50% surveillance sensitivity [94], although Nigeria reported cases from WPV transmission shortly after its publication [90]. In addition, other studies that used another different analytical approach to develop a silent circulation statistic for a hypothetical small population [95–97] considered surveillance quality varied from perfect (100% sensitivity) as well as 75%, 50%, and 25% and highlighted the impacts of different surveillance quality assumptions. However, this approach made some unrealistic assumptions that limited the overall inference of the results [98].

### Trends in polio surveillance activity

#### Utilization.

**Fig 2:**
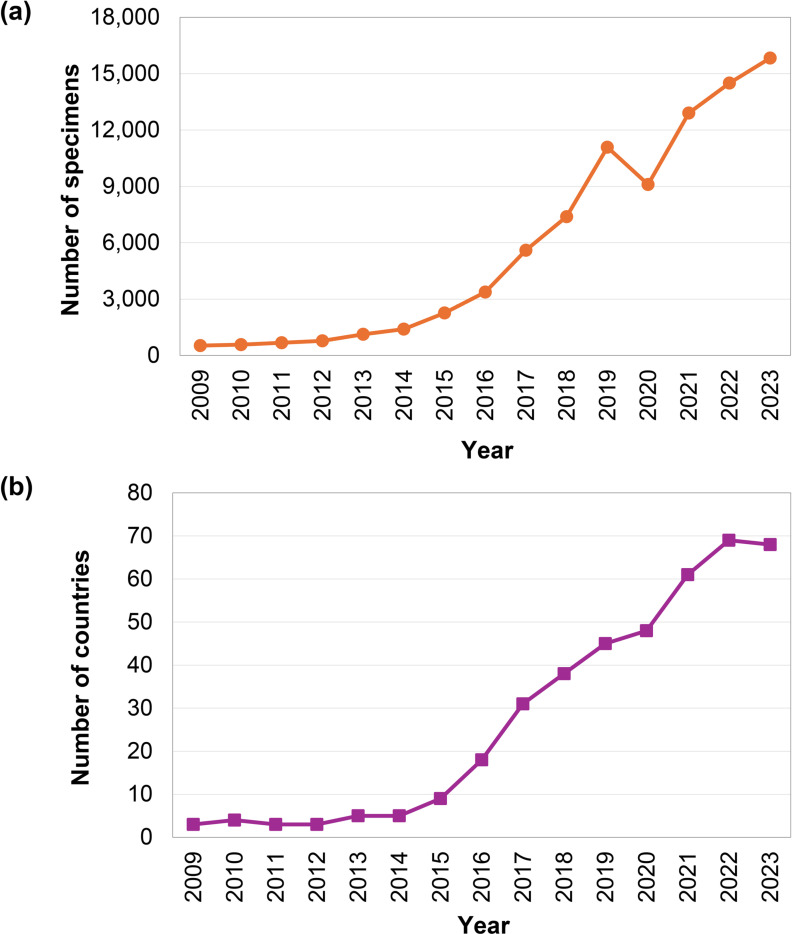
(a) Annual number of ES specimens and (b) the number of countries reporting ES reported by the GPEI.

While successive GPEI strategic plans anticipated that polio surveillance activities would ramp down and become integrated with other disease surveillance as polio eradication efforts succeeded [9], recent trends suggest otherwise. Fig 1 constructed based on data published in annual surveillance reports [22–33] shows that AFP testing has remained relatively stable over the past decade. During that time, with the introduction of IPV into routine immunization and ongoing poliovirus transmission, the adoption and expansion of ES led to substantial growth in laboratory processing of ES specimens (Fig 2a) and the number of countries performing and reporting ES (Fig 2b) based on data available in POLIS [36]. These trends suggest that poliovirus surveillance activities will likely continue at the current levels or potentially continue to expand (for ES) for the foreseeable future.

#### Time between AFP onset and official case reporting.

**Fig 3:**
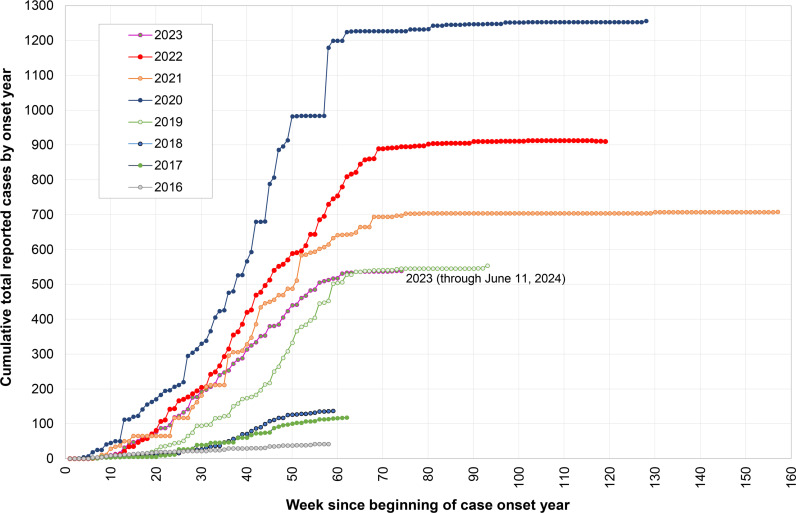
Cumulative distribution of polio cases (wild and circulating vaccine-derived of all types) reported each onset year by week reported since the beginning of the onset year (i.e., first epidemiological week in each calendar year counts as week 1) and extending for more than 53 weeks for cases reported in calendar years after the onset year.

Using data from POLIS [36], Fig 3 summarizes the time in weeks of reported confirmed cases caused by WPV and/or cVDPV with paralysis onset in the onset year indicated by the different colors. Since polio cases occur over time throughout a year, starting with week 0, reporting of these cases can begin within a few weeks. Thus, since cases with onset during the last week of December occur in the same onset year as cases with onset in early January, we should expect the curves to extend to around 56 or 57 weeks in the context of rapid specimen collection, transport, and processing. However, Fig 3 shows that GPEI reported small percentages of confirmed cases in most recent years well after 60 weeks. The actual impact of the extended time to notification remains unclear given that GPEI officially reports cases after notification by individual countries, which follows reporting from a national or GPLN laboratory to national health leaders. Thus, countries generally know about the detections and may act on them prior to GPEI reporting. For example, the detection and reporting for a case in New York with onset in June 2022 occurred in July 2022 (i.e., within 4 weeks) and led to an emergency effort to increase immunity with IPV oSIAs [72], despite the official GPEI reporting of the confirmed case on September 13, 2022 (i.e., 12 weeks after onset). In contrast, other types of delays may substantially increase the time to action. For example, if specimen collectors store samples to ship them in large batches, or if specimen transport and/or processing takes large amounts of time, then these increase the time to response according to the length of the delay(s).

#### Time between surveillance trigger and initiation of an oSIA.

For AFP cases caused by WPV1 and/or cVDPVs of any type with onset in each year between 2016–2020, Table 2 summarizes the number of distinct outbreaks as identified by GPEI with oSIAs and the average time between the onset of the first AFP case and the start of the first oSIA round. The process of deciding to conduct an oSIA in response to detected transmission includes many potential delays including specimen collection, transport, and processing, viral sequencing to identify the potential source as an importation or new emergence, confirmation of evidence of transmission, development of a formal request by national public health authorities to request stockpiled vaccine and/or financial resources (if needed), deployment of the stockpiled vaccine and/or financial resources (if needed), logistics, microplanning, communications, and other oSIA preparations. The implementation of coordinated cessation of type 2 OPV (OPV2) in mid-2016, and the associated increase in global adoption of IPV use in routine immunization, led to two subtle, yet substantial changes related to the detection of transmission. First, all detections of type 2 polioviruses required sequencing to inform decisions about circulation. As shown by modeling prior to OPV2 cessation, die out of the OPV-related viruses at the time of cessation takes time, with expected detection of some excretion and evolution and transmission of these viruses (i.e., VDPVs or ambiguous VDPVs) that may not indicate sustained transmission or emergence of a cVDPV [99–101]. Second, the introduction of IPV effectively increased the time between expected AFP cases in populations with live poliovirus transmission by providing protection from paralysis, but not effectively stopping or preventing transmission, which implied potentially longer times required for confirmation of transmission of outbreak polioviruses [100,102–104]. Increased time between AFP cases with IPV introduction provides some motivation for expansion of ES, which can potentially detect transmission in a population prior to (or in the absence of [41,105]) observing an AFP case.

Given expansion of ES and following evidence of the use of ES to trigger oSIAs, for 2021–2023, the results in Table 2 show the time to the first oSIA from the earliest surveillance signal (i.e., either onset of an AFP case or first detection from ES). The results in Table 2 show an overall trend toward an increase in the time between the detected event and the start of an oSIA. Although this may seem counterintuitive, because early detection in ES can support starting an oSIA prior to observation of a case, some time is required to fully characterize the phylogenetics and to determine that the signal indicates local transmission, and even after declaration of an outbreak, delays occur related to planning and/or obtaining resources (i.e., vaccine and finances) for the oSIA.

## Discussion

Prior GPEI strategic plans anticipated that successful global poliovirus eradication might lead to reduced requirements for financial investments for a poliovirus-specific surveillance system, and consequent transition of poliovirus surveillance capacity and resources into integrated disease surveillance systems [106]. However, as of 2024, polio eradication remains off track, and the need for active polio surveillance will likely continue “in perpetuity” as acknowledged in the recent GPEI strategic plan [21]. Ongoing development of new poliovirus vaccines [107,108] continues to increase the need for advanced intertypic differentiation and sequencing and an expansion of surveillance. In addition, innovations in genomic sequencing technologies generally, and for poliovirus specifically, may lead to substantial changes in the nature, timing, and investments in poliovirus surveillance methods [82,109]. The development and expansion of direct detection and genetic sequencing technologies [82–84,110,111] may create new opportunities for poliovirus surveillance system designs, which could prove disruptive to the existing system. GPEI recognizes the need to evaluate and implement new methods [21], as does its Independent Monitoring Board [112]. The expected growth by the GPEI (instead of decline) of poliovirus surveillance needs as of 2024, as well as innovations in laboratory technologies, create substantial uncertainty about the tradeoffs of different options and the future of poliovirus surveillance.

In contrast to the observed pattern of expansion over the past decade, we recognize that changes in policy or funding due to geopolitical movements or competing public health goals could lead to substantially decreased need and/or support for poliovirus-specific surveillance. In addition, national and global health leaders and institutions will likely continue to evaluate and make currently unforeseeable decisions about investments in poliovirus surveillance as surveillance technologies and resource availability change.

Several substantial uncertainties make forecasting the future of poliovirus surveillance challenging. GPEI transition plans assumed that the responsibility for poliovirus surveillance would revert to countries and that the GPLN would transition into an integrated disease surveillance system [21,113,114]. Activities to transition and integrate poliovirus-specific surveillance into broader disease surveillance continue to move forward, and this could potentially lead to a dramatic shift in the nature, conduct, and quality of poliovirus surveillance information in the near future. In addition, containment requirements could substantially reduce the number of laboratories able or willing to process poliovirus samples. For example, following global cessation of type 2 OPV in 2016, containment requirements for type 2 led clinical laboratories to end serological testing for type 2, which limited (censored) the information available related to ongoing excretion by a chronic iVDPV excreter after 2018 as highlighted by others [115]. Alternatively, demands for poliovirus-specific surveillance could expand. For example, ES could expand to additional areas, and/or research and development on new poliovirus vaccines could increase demands for serological analyses. The contrast between these extremes implies prospective model assumptions could poorly reflect the actual future performance, and they will likely need to undergo frequent re-evaluation.

A 2024 integrated modeling study assumed poliovirus surveillance will perform similarly to its current performance for the foreseeable future (2024–2035) and that high-income countries that use IPV-only will detect imported poliovirus transmission at the time of the first clinical case (if not before) [52]. These assumptions extend the timeline for degradation of surveillance quality such that it begins after the model time horizon (i.e., after 2035), pending further updating of assumptions about the nature and quality of prospective poliovirus surveillance. While IPV-only countries historically played a relatively minor role in the polio endgame, with no expected cases due to their achievement and maintenance of relatively high routine immunization coverage, but under-vaccinated subpopulations in these countries remain at risk for transmission [103,116].

Our focus on the use of poliovirus surveillance information for prospectively modeling policies and decisions implicitly ignores its value for monitoring trends and performing retrospective statistical analyses. We highlight this omission as a reminder of the scope of this analysis, because poliovirus surveillance plays a critical role in monitoring trends and evaluating the validity of national immunization coverage estimates. Poliovirus surveillance evolved substantially with the polio eradication endgame, from a system that distinguished polio cases from non-polio cases, then to also characterize the specific type of poliovirus that caused cases, then to also distinguish both the type and strain of poliovirus, and finally to provide genetic sequencing of the VP1 region of the poliovirus genome for positive AFP and ES samples, and now increasingly to provide whole genome sequencing data. Future evolution of poliovirus surveillance could shift entirely to whole genome sequencing, immunocompetency assessment of infected humans, and/or new methods to effectively trace environmental surveillance samples to the source(s) of infection. In addition, whenever polio eradication efforts end, the demand for active poliovirus-specific surveillance could substantially decline or disappear altogether. Key factors that will likely determine the future of poliovirus surveillance include the demand for specific information to support national, regional, and/or global policies, and the economics and trade-offs of surveillance (i.e., costs, benefits, and availability of financing).

Innovations in wastewater surveillance and ES for enteroviruses broadly (including polioviruses) could provide valuable tools for countries seeking to maintain surveillance for polioviruses, particularly as they shift away from OPV use, while also deriving greater benefit from the use of tools that offer information related to other pathogens (i.e., dual use or integrated disease surveillance systems). The expanded use of wastewater sampling to monitor SARS-CoV-2 viruses and their evolution substantially increased public demand for and recognition of population surveillance. As demonstrated by the experience with poliovirus surveillance, characterizing the health and economic benefits of expanded use of wastewater sampling and ES tools would likely benefit from health economic models that can account for the additional actionable information with respect to disease prevention and/or outbreak response that such innovations might imply.
